# Regulating AI-Based Medical Devices in Saudi Arabia: New Legal Paradigms in an Evolving Global Legal Order

**DOI:** 10.1007/s41649-024-00285-6

**Published:** 2024-06-21

**Authors:** Barry Solaiman

**Affiliations:** College of Law, Hamad Bin Khalifa University, Doha, Qatar

**Keywords:** Artificial intelligence (AI), Saudi Arabia, Medical devices, Data, Health, Law

## Abstract

This paper examines the Saudi Food and Drug Authority’s (SFDA) Guidance on Artificial Intelligence (AI) and Machine Learning (ML) technologies based Medical Devices (the MDS-G010). The SFDA has pioneered binding requirements designed for manufacturers to obtain Medical Device Marketing Authorization. The regulation of AI in health is at an early stage worldwide. Therefore, it is critical to examine the scope and nature of the MDS-G010, its influences, and its future directions. It is argued that the guidance is a patchwork of existing international best practices concerning AI regulation, incorporates adapted forms of non-AI-based guidelines, and builds on existing legal requirements in the SFDA’s existing regulatory architecture. There is particular congruence with the approaches of the US Food and Drug Administration (FDA) and the International Medical Device Regulators Forum (IMDRF), but the SFDA goes beyond those approaches to incorporate other best practices into its guidance. Additionally, the binding nature of the MDS-G010 is complex. There are binding ‘components’ within the guidance, but the incorporation of non-binding international best practices which are subordinate to national law results in a lack of clarity about how penalties for non-compliance will operate.

## Introduction and Background

The development of laws and regulations governing the use of artificial intelligence (AI) in healthcare is at a nascent stage. A four-stage paradigm has arisen in recent years.^1^ First, while AI has been around for many decades, it now presents new challenges for regulators that ‘traditional’ technologies do not, such as the autonomous and evolving nature of AI algorithms. Second, lawyers seek to adapt existing laws to deal with those challenges but find that the process only takes us so far. Third, in the meantime, soft law consisting of principles and guidelines is developed as a stopgap. Finally, new hard law is created.

One key area of focus within this paradigm is medical device regulations, which are crucial in the health space because AI usually manifests itself in medical practice through these devices. However, existing regulations have not explicitly addressed the unique challenges posed by AI-based medical devices (medical devices that incorporate AI).^2^ The regulations are often premised on devices that use ‘locked’ algorithms. These devices will produce the same result every time they are used, compared to devices using ‘adaptive’ AI algorithms that may produce different results every time they are used (FDA 2021c, 3). The underlying logic of locked algorithms is usually also straightforward and understood. In contrast, AI decisions may be unexplainable owing to the numerousness and complexity of the neural network underpinning an AI system (the black box problem) (Babic et al. 2021; Solaiman and Bloom 2022).

As the number of AI-based medical devices grows, dealing with these challenges will become ever more crucial for medical device regulators worldwide. The FDA (n.d.) has approved more than 150 such devices in recent years but has used the existing regulatory pathways described later in this paper, which are not designed to deal specifically with the challenges of AI. Nevertheless, the US FDA is at the forefront of discussions to regulate such devices and has published non-binding guidance. The European Union (EU) is also building on existing medical device regulations to make additional requirements for authorising AI systems through its proposed Artificial Intelligence Act (AI-Act) (European Parliament 2024). That approach will be limited because the additional requirements do not address matters of health directly relevant to AI-based medical devices. Of the emergent approaches, the Kingdom of Saudia Arabia (KSA), through the Saudi Food and Drug Authority (SFDA), has taken a leading role in establishing and implementing guidelines specifically for AI-based medical devices. It is this regime which is examined in this paper.

The SFDA’s developments have not evolved in a cocoon. They are linked to broader global developments that are important to unpack. Nevertheless, they are among the first in the world and now would be an opportune moment to examine the SFDA’s approach owing to the dearth of literature. It is also important to note that the SFDA’s developments are part of a regional focus on developing governance structures for AI and related matters. Qatar was the first country in the Middle East to create a data protection law, which has important implications for AI in health (Solaiman 2023a).^3^ The emirates of Abu Dhabi and Dubai in the United Arab Emirates (UAE) have been among the first countries in the world to develop regulations for the use of AI in health generally (Department of Health 2018; Health Informatics and Smart Health Department 2021, 1). Saudi has taken on a leading role in AI medical device regulations.^4^

There are three parts to the analysis below. First, the paper explores the nature and scope of the SFDA’s regulatory architecture for AI-based medical devices. Second, it situates that architecture within the global order, particularly by reference to the USA and South Korea, that the SFDA’s documentation refers to and builds on, and the EU. Third, it considers future implications of the SFDA approach. The aim here is not to appraise the specific requirements of the SFDA regulations nor provide normative arguments about how the regulations ought to develop.^5^ At this stage, it is difficult to make significant normative claims because information about the effect of the regulations in practice is lacking. More time will be needed to accrue such experience. Thus, overall, it will be seen that the SFDA has developed a detailed and complex ecosystem of regulations for AI-based medical devices.^6^ Its efforts are pioneering, but further research will be needed to appraise the success of these regulations once experience has built from their implementation.

## The SFDA’s Regulatory Architecture

It is helpful first to outline the regulations in KSA that are relevant to medical devices generally. These are outlined as a reference point to the relevant guidance documents discussed in this paper, and to illustrate the scope of the SFDA’s authority.

Table 1 elucidates the significant developments in KSA since 2020 in medical device regulations. While the AI-specific guidance did not arise until 2022, it should not be read in isolation because it is a connected regulatory ecosystem. To unravel this environment, the section below begins with an analysis of the ‘Guidance on Artificial Intelligence (AI) and Machine Learning (ML) technologies based Medical Devices’ (MDS-G010) that regulates AI-based medical devices and builds out the analysis from there, highlighting its relation to other instruments. It has been noted elsewhere that ‘only the Saudi Food & Drug Authority had published an enforceable guidance establishing regulatory requirements for AI/ML in medical devices’ (Henry and Thiel 2022). An important query in the passages below is the degree to which guidelines are actually binding because this could be a distinguishing feature of the SFDA’s regulations when compared to other jurisdictions.

**Table 1:** Regulations for Medical Devices in Saudi Arabia

Regulation	Version number	Date
Guidance on Software as a Medical Device^a^	(v.1.0)	9 April 2018
Guidance on Requirements for Medical Device Listing and Marketing Authorization’ (MDS G5)^b^	(v.5.0)	22 June 2020
Implementing Regulation of the Law of Medical Devices^c^	(N/A)	26 September 2021
Requirements for Medical Devices Marketing Authorization (MDS-REQ 1)^d^	(v.6.0)	19 December 2021
Requirements for Unique Device Identification (UDI) for Medical Devices (MDS-REQ 7)^e^	(v.4.0)	24 May 2022
Requirements for Inspections and Quality Management System for Medical Devices (MDS-REQ 10)^f^	(v.1.0)	22 June 2022
Requirements for Licensing of Medical Establishments (MDS-REQ 9)^g^	(v.1.0)	31 July 2022
National Diagnostic Reference Levels (MDS G8)^h^	(v.2.0)	26 October 2022
Guidance on Artificial Intelligence (AI) and Machine Learning (ML) technologies based Medical Devices’ (MDS-G010)^i^	(v.1.0)	29 November 2022
Requirements for Clinical Trials of Medical Devices (MDS-REQ 2)^j^	(v.4.1)	26 December 2022
‘Guidance for Points of Care (POC) Medical Devices Manufacturing’ (MDS G009)^k^	(v.1.0)	8 January 2023
Requirements for Post-Market Surveillance of Medical Devices (MDS-REQ 11)^l^	(v.2.0)	23 March 2023
‘Requirements for Safe Use of Medical Devices Inside Healthcare Facilities (MDS-REQ 3)^m^	(v.2.0)	15 May 2023

^a^MDS-G23: SFDA, Guidance on software as a medical device (9 April 2018) v.1.0

^b^MDS-G5: SFDA, Guidance on requirements for medical device listing and marketing authorization (22 June 2020) v.5.0

^c^IRMD: SFDA, Implementing regulation of the law of medical devices (26 September 2021) Issued by SFDA Board Decision No. (3–29-1443) dated 19/02/1443 AH

^d^MDS-REQ 1: SFDA, Requirements for medical devices marketing authorization (19 December 2021) v.6.0 MDS-REQ-001-V6/211219

^e^MDS-REQ 7: SFDA, Requirements for unique device identification (UDI) for medical devices (24 May 2022) v.4.0 MDS-REQ-007-V4/220524

^f^MDS-REQ 10: SFDA, Requirements for inspections and quality management system for medical devices (22 June 2022) v.1.0 MDS-REQ-010-V1/220622

^g^MDS-REQ 9: SFDA, Requirements for licensing of medical devices establishments (31 July 2022) v.1.0 MDS-REQ-009-V1/220731

^h^MDS-G008: SFDA, National diagnostic reference levels (26 October 2022) MDS-G-008-V2/230101

^i^MDS-G010: SFDA, Guidance on artificial intelligence (AI) and machine learning (ML) technologies based medical devices (29 November 2022) v.1.0 MDS-G-010-V1/230103

^j^MDS-REQ 2: SFDA, Requirements for clinical trials of medical devices (26 December 2022) v.4.1 MDS-REQ-002-V4.1/221226

^k^MDS-G009: SFDA, Guidance for points of care (POC) medical devices manufacturing (8 January 2023) v.1.0 MDS-G-009-V1/230108

^l^MDS-REQ 11: SFDA, Requirements for post-market surveillance of medical devices (23 March 2023) v.2.0 MDS-REQ-011-V2/230323

^m^MDS-REQ 3: SFDA, Requirements for safe use of medical devices inside healthcare facilities’ (15 May 2023) v.2.0 MDS-REQ-003-V2/2305308

## The MDS-G010: Regulating AI-Based Medical Devices

The purpose of the MDS-G010 is to clarify the requirements for obtaining Medical Device Marketing Authorization (MDMA) for AI/ML-based medical devices to place them on the market in KSA (MDS-G010, 3). Medical devices may only be marketed following registration and obtaining marketing authorisation from the SFDA under the Law of Medical Devices (IRMD, art 8). AI devices are those ‘that diagnose, manage or predict diseases by analyzing medical data’ (MDS-G010, 3). To achieve its objectives, the MDS-G010 creates classification criteria and control methods for AI-based medical devices. The key question is what the product developer (manufacturer) intends the device to be used for (Babic et al. 2021; Solaiman and Bloom 2022).^7^ If it is intended that the device is used for ‘investigation, detection diagnosis, monitoring, treatment, or management of any medical condition, disease, anatomy or physiological process’, then the device will be considered as a medical device, and regulatory controls will apply. The device can be ‘any instrument, apparatus, implant, in vitro reagent or calibrator, software, or material used for operating medical devices, or any other similar or related articles, intended to be used alone or in combination with other devices’ (European Parliament 2024). Those determinations are made by examining the product specifications, instructions for using the device, and any other information provided by the product developer.

While not exhaustive, the SFDA provides examples of devices that may be regulated under the regulation. This includes in vitro diagnostic tools using AI to identify and quantify different types of cells or AI-based biosensors that can predict the probability of disease. This approach is congruent with the European Union’s (EU) Artificial Intelligence Act (AI-Act). That regulation stipulates that AI-based devices subject to other EU regulations must comply with those regulations and the requirements of the AI-Act (European Parliament 2024, art 43(3)). The AI-Act is not specific to healthcare, but it includes both the Medical Device Regulation (MDR) and the Regulation on In-Vitro Diagnostic Medical Devices (IVD) in its annexes.^8^ This inclusion means those devices must comply with the MDR or IVD and the AI-Act requirements.

The SFDA in Saudi Arabia envisages similar devices falling within its regulatory remit and follows a similar regulatory structure to the EU. AI-based in-vitro devices must first be approved under the Law of Medical Devices and must then additionally comply with the MDS-G010 requirements. However, manufacturers must comply with diverging requirements for the additional requirements in both regimes. The AI-Act requirements are based on a risk classification structure that differs from the MDS-G010 requirements of the SFDA. Thus, while the initial medical device or in-vitro diagnostic device compliance requirements will be generally familiar, the bolt-on requirements of the latter regulations will not be. This will make navigating both markets complex and time-consuming for manufacturers.

### Classification Criteria and Control Methods

The MDS-G010 sets out the classification criteria and control methods for AI medical devices. First are the ‘Premarket Review Considerations’. The manufacturer must comply with technical documentation depending on whether the device is a ‘medical device’ or an ‘in-vitro diagnostic medical device’ (MDS-REQ 1, 4, 50 & 59). Again, these requirements sit apart from the AI component of a device. In general, the documentation requires a description of the device, design and manufacturing information, essential principles of safety and performance, a risk–benefit analysis, and a post-market surveillance plan (MDS-G010, 5). Underlying those areas are substantive, detailed, and granular requirements. For example, the medical device technical documentation requires a description of materials that have direct or indirect contact with the human body, the purpose of the device for patients, explanations of novel features, instructions in both English and Arabic, technical drawings, voltages, laboratory test results, and many others (MDS-REQ 1, Annex 3).

Moving from the general to the specific, ‘special consideration’ must also be given to the risks associated with the device, its intended purpose, and anticipated use in the digital health system (MDS-G010, 5). Documentation that demonstrates performance testing should provide a usability study verifying the user has been provided with information to connect to the device and ensure that a connection has been made correctly (European Parliament 2024). In this regard, the MDS-G010 introduces requirements more relevant to digital AI devices where the anticipated use of the AI device within the digital health system and how users connect to that system must be contemplated.

Two matters are unclear here, though. First, the ‘digital health system’ is a vague and undefined term. Indeed, the term is used only once in the guidance. Does the ecosystem mean broadly the market for AI medical devices, or does it mean the hospital ecosystem or something else? Second, it is striking that the ‘user’ is never defined in the MDS-G010. This definition is critical for determining who the law is meant to protect. One can look to the other guidelines in Table 1 for potential answers, but that approach is unclear.

Presumably, the definition follows that of the IRMD, 3, which defines a user as ‘A person, whether a professional, non-professional, or patient, who uses a medical device or supply’. ‘Users’ are similarly defined in MDS-REQ 3, 13 as ‘A person, whether a professional, lay person or a patient, who uses a medical device’. Also, MDS-REQ 10, 20 defines a user as ‘A person, whether a professional, non-professional, or patient, who uses a medical device or supply’. Other guidelines in Table 1 are not as clear, using the following phrases: ‘intended users of the device’; ‘the user or clinician’ (both MDS-G5, 73); ‘the means of protecting the patient, user, or other person’; ‘the user and/or patient’ (both MDS-G5, 80); ‘healthcare providers and users’ (MDS-REQ 11, 11); ‘patient, user or other person’ (MDS-REQ 2, 19). A consistent definitional approach is needed across this ecosystem to clarify what a user means. This is important for establishing clear lines of accountability.

### Harmonization with Existing Approaches?

A significant part of the MDS-G010 is the clinical evaluation criteria. The SFDA notes that there is no internationally agreed framework concerning such criteria for AI medical devices but, nevertheless, attempts to set out detailed expectations concerning device safety, effectiveness, and performance before AI medical devices can be placed on the market (MDS-G010, 6).

In 2018, the SFDA adopted the principles related to ‘Software as a Medical Device’ (SaMD) agreed upon by the International Medical Device Regulators Forum (IMDRF) (MDS-G23, 2). SaMD is defined by the (IMDRF) as ‘software intended to be used for one or more medical purposes that perform these purposes without being part of a hardware medical device’ (IMDRF SaMD Working Group 2013, 6). This includes in-vitro diagnostic (IVD) medical devices. The SFDA uses those principles as a basis for the clinical evaluation of AI medical devices by requiring a ‘valid clinical association, analytical/technical validation, and clinical validation’ (MDS-G010, 7). The SFDA is also inspired by other documents of the IMDRF. Namely, key definitions of medical devices agreed by the IMDRF in 2022, a possible framework for risk categorisation, and guidance on quality management systems (MDS-G010, 22). It also refers to guidelines from the Ministry of Food and Drug Safety (MFDS) in South Korea on reviewing and approving AI-based medical devices and the Food and Drug Administration’s (FDA) proposed regulatory framework on AI-based medical devices.

The pool of inspiration is, therefore, necessarily small. The IMDRF documents are not (yet) tailored to AI-based medical devices specifically, aside from the definitional documentation. Nevertheless, there appears to be some alignment in SFDA, FDA, and MFDS approaches. The FDA’s guidelines consist, among other things, of three important documents: the ‘Action Plan’ (FDA 2021a), the ‘Proposed Regulatory Framework’ (FDA 2019a), and the draft ‘recommendations for a Predetermined Change Control Plan for Artificial Intelligence/Machine Learning (AI/ML)-Enabled Device Software Functions’ (FDA 2023a). The FDA (2021b) notes that its ‘Proposed Regulatory Framework’ builds on its own frameworks and programs and relies on ‘the IMDRF’s risk categorization principles’. Similarly, the MFDS’s guidance in South Korea has also sought harmonisation with the IMDRF’s principles (National Institute of Food and Drug Safety Evaluation 2022, 2). It is likely that, in citing the FDA’s ‘proposed regulatory framework’, South Korea’s guidance, and the IMDRF documentation, the SFDA was either following the approach of the FDA and MFDS, or there was some level of coordination or alignment. This process highlights how AI regulation is a newly emerging area without clear precedent. Regulators are looking to build on existing regulations and tailor them to AI.

For now, in the USA, AI-based medical devices are approved through a ‘de novo’ pathway, a premarket approval, or the 510(k) pathway, which allows clearance for a medical device if it is substantially equivalent to a device that has been formerly cleared (a predicate). Muehlematter et al. (2023) note that ‘substantial equivalence’ has been leniently interpreted. This has led to a ‘predicate creep’, with generations of devices cleared for their substantial equivalence to previous devices despite having iterative design changes ‘resulting in devices dissimilar from original predicates’. The FDA (2019b) states that a device is ‘substantially equivalent’ to a predicate device if it has the same intended use of the predicate and the same technological characteristics, or has the same intended use and different technological characteristics, does not raise different questions of safety and effectiveness, and the information submitted to the FDA demonstrates the device is as safe and effective as the legally marketed device.

Muehlematter et al. (2023, e620) analysed all approved devices through the 510(k) pathway between 2019 and 2021. Of the devices approved, 83.2% were approved for radiology, 9.1% for cardiovascular uses, and a plethora of other areas for the remaining approvals. Approximately 59% of AI-based devices examined were approved based on having substantial equivalence with devices that had an underlying AI technology. For approximately 8% of devices, it was unclear whether the predicate device could qualify as an AI device. A significant 33% of AI-based devices examined were approved on the basis of non-AI-based medical devices. However, the intended use between a new device and its predicate can differ, despite receiving approval from the FDA under its definition of substantial equivalence (Muehlematter et al. 2023, e623).

Muehlematter et al. (2023, e623) provide a summary of examples, including a radiological device cleared for assessing breast abnormalities in MRI data subsequently serving as a predicate for one AI device examining brain tissue abnormalities in CT scans, a second device detecting pulmonary nodules in CT image data, and a third device examining coronary heart disease — highlighting how the intended use can vary somewhat. Another example was of a radiological device cleared in 2019 for visualising regions and organs of the body at risk and potentially requiring treatment involving radiation. That device was cleared based on a predicate that was used for a similar intended purpose, but the new device implemented AI for its analysis, whereas the predicate did not (Muehlematter et al. 2023, e624). Ultimately, the concern is that the FDA is using broad definitional interpretations at its discretion which is favourable to industry practices which raises safety concerns (Muehlematter et al. 2023, e623-e624).

As such, the regulatory approach in the USA builds on existing rules for clearing non-AI-based devices rather than approving such devices through a specific ‘AI’ pathway. Furthermore, it should be noted that the FDA’s ‘Action Plan’ and the ‘Proposed Regulatory Framework’ are non-binding documents, while the ‘Change Control Plan’ is only in draft form. Similarly, South Korea’s guidance is also non-binding.

Placing the SFDA’s approach within the broader ecosystem is not straightforward. While the Law of Medical Devices is binding because it is supported by sanctions and penalties (IRMD, art 42), it is not clear whether the other instruments listed in Table 1 are binding or not. The likelihood is that MDS-G010 is non-binding itself because sanctions are not stipulated, and the SFDA appears to align with USA and South Korean approaches. However, the MDS-G010 does refer to binding requirements in the Medical Device Law. Consequently, a manufacturer may be subject to binding ‘components’ of the MDS-G010 and other documents. That is to say, the components of the guidelines that refer to the law may be binding and subject to the underlying instrument. Examples of this are highlighted in the section below where relevant.

Additionally, the ‘clinical evaluation’ requirements set out below contemplate evaluations ‘based on a comparator device’, noting that the manufacturer ‘must demonstrate sufficient clinical and technical equivalence of the other device’ (MDS-G010, 9). Where equivalence cannot be demonstrated, then other evidence must be used. One query about these requirements is whether they replicate the US predicate pathway above, whereby a non-AI device can be used as a predicate, or whether these are separate requirements mandating that the predicate device is an AI-based medical device. The guidance is not clear on these points.

#### Clinical Evaluation

It is helpful to delve deeper into the requirements of the MDS-G010 to understand more about the SFDA’s approach compared to other regulators. It should be noted that reference to the IMDRF principles only bears relevance in the MDS-G010 once the ‘Clinical Evaluation’ criterion is set out. Manufacturers must generate evidence to demonstrate (a) a valid clinical association, (b) analytical/technical validation, and (c) clinical validation of the AI-based medical device.

To demonstrate a valid clinical association between the output of the AI device and the targeted clinical condition, evidence should demonstrate that the output is ‘clinically accepted based on existing evidence in published scientific literature, original clinical research, and/or clinical guidelines’.^9^ This evidence is needed to demonstrate the relevance of the data to the problem in clinical practice and to establish that the intended use of the AI medical device aligns with that problem. The manufacturer must generate new evidence where evidence cannot be provided from existing sources. The SFDA suggests this can be achieved through a secondary data analysis or clinical trials and expects that AI medical devices will often require such new evidence. This is because the evidence supporting AI-based medical devices is ‘immature’, meaning that confidence in the evidence will be low. The SFDA states that medical devices ‘often will be classified as having novel clinical association as these devices may involve new inputs or outputs, novel algorithms, new intended target population, or new intended use’.

It is unclear what evidence the SFDA relied on to determine that AI-based medical devices will ‘often’ be classified as ‘novel’. As noted above, experience in the US shows that approximately one-third of devices were cleared based on non-AI-based medical devices. While the intended use of the AI device and its predicate can differ, the extent to which that difference means ‘novelty’ is unclear. There were cases where the new AI-based device was similar to the predicate, with the main difference being the addition of AI to the device for automation purposes (Muehlematter et al. 2023, e624). This at the very least raises questions about the extent to which the device is ‘novel’ and how such ‘novelty’ should be defined. Furthermore, the FDA’s de novo pathway exists to clear devices that ‘represent novel technologies in contrast to 510(k)-cleared devices’ (Muehlematter et al. 2023, e618). However, only a quarter of devices that were cleared in Muehlematter’s (2023, d625) analysis originated in a de novo cleared device across all generations. The pathway for ‘novel’ devices was thu not the primary route through which clearance has been given for AI-based medical devices in the USA. Experiences may differ, but in the USA at least where the market is larger, there are indications that the picture is more complex than the SFDA paints. Determining the ‘novelty’ of a device will require a complex assessment of factors concerning the underlying technology of the device seeking clearance, based on clearly defined criteria of what novelty means. It will be helpful if the SFDA revisits its analysis of novelty and implements clearer rubrics about the circumstances in which AI devices will be classified as being novel.

The SFDA may also seek to distinguish itself from the FDA. It has been noted how devices approved on the predicate pathway changed frequently along the device’s predicate network, which raised safety concerns. A ‘predicate creep’ may ensue, whereby successive iterative design changes may result in unproven devices being approved as increasingly dissimilar predicates. The SFDA may face a potential dilemma where manufacturers present evidence that they submitted to the FDA and were deemed satisfactory for the FDA based on its pathways. Should the SFDA accept that evidence sufficient for the FDA is sufficient for its requirements, or should the SFDA do further analyses to determine whether those devices are actually iteratively different from predicates, such that they may not be approved? The SFDA may have an opportunity to raise standards for protection by differentiating its approach.

The following requirement under the MDS-G010 is that the manufacturer demonstrate analytical/technical validation. This evaluates the correctness of the input data processing by the device to create a reliable output (MDS-G010, 7). Evidence should be provided to establish that the device requirements have been fulfilled and demonstrate that the device meets the specifications for its intended use. That evidence is expected to be generated as part of the ‘quality management system’ (QMS) protocols concerning ‘verification’ and ‘validation’ that use labelled reference datasets. A QMS is a formalised system that documents processes, procedures, and responsibilities for achieving policies and objectives (American Society for Quality, n.d.). It arises from the International Organization for Standardization (ISO) 9001, which is a globally recognised standard. The ISO is an independent organization consisting of national standards bodies that set global standards in a range of technical and non-technical fields. The stipulation concerning the QMS may refer to a binding ‘component’. The MDS-REQ 10 covers QMS requirements, including obtaining a QMS certificate from the SFDA (MDS-REQ 10, 5). That guidance is supported by penalties where the provisions are violated (MDS-REQ 10, 18). To underscore this binding requirement, the Law of Medical Devices requires that technical documents be submitted, including those for verification and validation of the product, including clinical trials (IRMD, art 10). That is also backed by penalties for non-compliance (IRMD, art 42).

The final requirement for clinical evaluation under the MDS-G010 is that the manufacturer should demonstrate clinical validation. This measures the ability of the device to ‘yield a clinically meaningful outcome associated to the intended use of the device’, taking into account the target population (MDS-G010, 8). This is demonstrated both pre-market and post-market by providing data from studies for devices created for the same intended use, extrapolating data from studies for devices that were not of the same intended use or where both are lacking, by generating new clinical data.

As with other provisions, this part of the guidance incorporates the IMDRF guidance on the metrics that may be used to determine clinical validation, such as positive predictive value (PPV), negative predictive value (NPV), likelihood ratio negative (LR-), likelihood positive ratio (LR +), and clinical usability. These metrics assist in measuring whether the device is working as intended. An independent review may be carried out of the results of a clinical evaluation to assess whether the device is ‘clinically meaningful to users’ (MDS-G010, 9).

The Saudi guidance then introduces a novel approach to determining the minimum standards and good practice for clinical evaluations. The guidance notes that there are no international standards for clinical evaluations, so the SFDA partially adapts standards from the World Health Organization (WHO) — something that neither their counterparts in the USA nor South Korea explicitly include in their guidance. The partially adapted WHO elements appear to be worded recommendations. Thus, some provisions state, for example, that manufacturers *should* ‘assess whether the promised medical benefit is achieved is consistent with the state of the art’. ‘Should’ is the predominant term used, but some provisions also ‘advise’ the manufacturer to ‘evaluate user and system elements’ by considering a range of factors (MDS-G010, 9-10). It is also likely that this ‘component’ of the MDS-G010 is a non-binding section because it incorporates non-binding guidance from the WHO.

Nevertheless, incorporating more specific AI-relevant guidelines indicates a potential path for developing AI medical device regulations elsewhere. Other incorporated WHO standards include recommendations that manufacturers should generate evidence on the device’s performance that can be generalised to the intended population by conducting multisite clinical investigations and analysing the model on appropriate subgroups (MDS-G010, 11). The AI device should also be evaluated in clinical settings, and the effects of their studies on healthcare organisations should be considered. There is also some crossover with the ‘lifecycle’ approach to regulation highlighted by the FDA when the MDS-G010, 12 states that ‘manufacturers are required to use post-market continuous monitoring of safety, effectiveness, and performance’ in real-world settings because of the ability of AI to learn continuously.

#### Risk Management

The risk management criteria explicitly note that AI medical devices may pose risks that ‘could jeopardize patient health and safety, increase inequalities and inefficiencies, undermine trust in healthcare, and adversely impact the management of healthcare’ (MDS-G010, 13). Here, the MDS-G010 leans into the MDS-REQ 1 on the Requirements for Medical Devices Marketing Authorization, which requires that manufacturers demonstrate their medical device does ‘not pose unacceptable risks, and that the benefits of their intended use outweigh the overall residual risk’. The MDS-REQ 1, 3 itself specifies the requirements for obtaining medical device marketing authorisation under the Medical Devices Law. A risk management plan should be adopted to demonstrate that unacceptable risks are not posed. Since the MDS-REQ 1 does not explicitly address AI medical devices, the MDS-G010 guidance in this section may be considered as an add-on to the MDS-REQ 1 requirements where AI devices are involved. In this manner, the ‘risk management’ component of the MDS-G010 could be seen as a binding component of the regulations because it flows from the requirements of the Medical Devices Law.

In this section of the MDS-G010, the SFDA also refers to ISO 14971/2019, which also shows alignment with the FDA. The secretariat for ISO 14971/2019 was the American National Standards Institute (ANSI) of the USA, which facilitates and coordinates voluntary standards and conformity assessment systems.^10^ That ISO ‘specifies terminology, principles and a process for risk management of medical devices’ and is designed to assist manufacturers to identify hazards, estimate and evaluate associated risks, control the risks, and monitor the effectiveness of those controls (ISO 2019). While useful for medical devices, the ISO does not apply to AI-based medical devices. As such, the Association for the Advancement of Medical Instrumentation (AAMI) (another non-profit organization that is a primary source for consensus standards) provided guidance on applying ISO 14971/2019 specifically to AI medical devices (AAMI 2021). The SFDA cites that guidance in the risk management section of the MDS-G010 (published in 2022). In early 2023, the FDA recognized the same guidance for the risk management of AI devices (FDA 2023b; Stallard 2023).

The MDS-G010 notes the ‘elevated risks’ posed by AI ‘around data management, feature extraction, algorithm training, model evaluation, and cyber and information security’ (MDS-G010, 13). There could be risks to patient safety caused by wrongful inferences in the data, harmful recommendations, or correlations being drawn instead of causation. Consequently, data scientists should be involved in the risk management team. A risk management plan should include responsibilities for personnel, thresholds for risk acceptability, methods used to evaluate risks, interoperability risks, and cybersecurity. The risk analysis should query whether the AI system provides treatment or diagnostic recommendations, what the target population is of the device in terms of the seriousness of the patient condition, whether errors are detectable, whether the device can adjust its performance over time, and others (MDS-G010, 14). In this manner, the requirements specifically contemplate the risks of AI medical devices instead of general medical devices.

The MDS-G010 also recommends that a review, according to Clause 9 of ISO 14971/2019, be performed before the commercial release of the AI-based medical device (MDS-G010, 15, referencing AAMI 2023). One point to highlight regarding ISO 14971 is that it is technically a voluntary standard subordinate to national law. Considering that the risk management requirements under the MDS-G010 are likely an add-on to the MDS-REQ 1, which itself sets out the legal requirements of the Medical Devices Law, the inclusion of the ISO standards is technically an inclusion of non-binding standards. However, the FDA also recognises the ISO and its standards will likely be considered as best practices for manufacturers to adhere to.

Ultimately, the explicit recognition that AI medical devices can pose unacceptable risks to patient health and safety and the associated requirements to develop a risk management plan are rarely found in medical device regulations globally. It is a positive development that in the absence of broad international standards, some agreed standards, such as those from ISO 14971, are filtering through to the requirements in the guidelines at a national level. These developments are recent, though, and further research will be needed to determine exactly how these requirements manifest in practice and what the strengths and limitations will be for risk management as time progresses.

#### Quality Management System and Change Notification

The final two areas covered by the MDS-G010 similarly build on existing legal approaches. Thus, the provisions on QMS state that AI medical devices shall be designed and manufactured in accordance with ISO 13485 (Medical Devices Quality Management System) (ISO 2016). ISO 13485 is not specific to AI, and it appears that neither the FDA nor South Korea’s MFDS has incorporated it into their guidance, whereas the SFDA has. These ISO requirements are, therefore, adapted by the SFDA to include AI devices.

The designer and deployer are responsible for implementing the QMS. The QMS covers ‘developing a quality policy, quality objectives, procedures, and project-specific plans that are customer focused’ (MDS-G010, 16). To achieve this objective, there must be appropriate resources and personnel ‘in meeting SFDA regulation’, and the personnel must be competent in both AI and ‘clinical aspects of the use of the software’. Infrastructure must be in place and available throughout the AI lifecycle processes to support the development, production, and maintenance of the AI device. There are also requirements concerning the traceability of AI, measurement and monitoring (including post-market surveillance like tracking complaints), and evaluation of AI processes. Therefore, the focus of QMS is squarely on AI systems. The stipulation that QMS must be implemented to meet the requirements of the SFDA regulation highlights another binding component of the AI guidelines that flows from the Medical Device Law.

There are also requirements concerning change notification. These do not specifically address AI but restate the MDS-REQ 1, 5 requirements for medical device marketing authorisation. There is a requirement to inform the SFDA of any significant or non-significant change for major occurrences that could ‘could reasonably be expected to directly affect the safety or effectiveness of a device’ or minor occurrences that could have the same effect ‘indirectly’. The manufacturer must have procedures within the QMS for evaluating changes and informing the SFDA of those changes (MDS-G010, 18).

This places significant responsibility on developers to determine the significance of changes on the safety and effectiveness of a device. However, AI may present greater challenges because of its adaptive nature, so more detailed guidance and assistance may be needed for manufacturers regarding regulatory expectations in this context. The MDS-G010 merely states that manufacturers must have ‘procedures’ in place but it is questionable whether that requirement will suffice. The Medicines and Healthcare Products Regulatory Agency (MHRA) in the United Kingdom (UK) plans to produce specific guidance for change management of SaMDs, and this may be an area that the SFDA looks to in the future to improve its guidance (Medicines & Healthcare products Regulatory Agency 2023). This is particularly important for change notification because, like the QMS, the change notification requirements are also a binding component of the guidelines since they build on binding requirements in the regulatory scheme. Manufacturers are bound by those requirements, so they should have clearer guidance in place for compliance in the AI context.

## Conclusion: Moving Forward

The SFDA’s Guidance on Artificial Intelligence (AI) and Machine Learning (ML) technology-based Medical Devices (the MDS-G010) is an attempt to piece together various best practices to regulate AI-based medical devices. In the absence of internationally agreed standards or an existing comprehensive model to regulate this space, the SFDA has attempted to lay a foundation that other regulators should consider carefully. This article has examined the nature and scope of the regulation. It has also highlighted the patchwork of best practices incorporated into the guidelines and situated these developments within the broader regulatory environment.

On the nature and scope, the MDS-G010 focuses specifically on best practices related to AI-based medical devices and the protections needed for patient care — something that is lacking in the global regulatory landscape. The binding nature of those regulations is unclear. On the one hand, the MDS-G010 incorporates international non-binding best practices. On the other hand, various provisions are binding ‘components’ of the overall regulatory architecture because they build on other binding SFDA requirements and must be seen as add-ons to those. Non-adherence is backed by penalty and enforcement provisions in the underlying law. For clarity, it would be helpful if future versions of the MDS-G010 definitively state whether it is binding or not.

The regulatory parallels with other systems and the sphere of influence in the MDS-G010 are important for situating the SFDA’s approach in the broader scheme. It is doubtful that the EU’s AI-Act influences the MDS-G010, but a parallel was noted. Namely, manufacturers must adhere to the underlying medical device or in-vitro diagnostic device regulations and then also adhere to the additional requirements concerning AI. For the EU, the additional requirements are in the AI-Act, whereas they can be found in the MDS-G010 in Saudi (two very different regulations).

The SFDA is influenced by the IMDRF, the US FDA, and the Korean MFDS, having cited them in their guidance. There is harmonisation in all three countries in following the IMDRF, which signals that it will be central to other regulators creating regulations in this area in the future. There is also alignment between the SDFA and FDA in the recognition of ISO 14971/2019 for the purposes of regulating AI-based medical devices. However, the SFDA was the first to act by incorporating the requirements of the ISO into the MDS-G010. The SFDA is also ahead of other regulators by adapting ISO 13485.

The MDS-G010 is a recent development, so time and experience will be needed to accrue data about the practical implementation and implications for manufacturers on the ground. For example, it was queried above whether the SFDA’s assumption that devices will ‘often’ be classified as being ‘novel’ is true given the FDA’s experience, and data will be needed about whether the SFDA’s experience has diverged from the FDA. Once that data arises, it will be crucial to revisit the regulation, examine the successes and challenges, and determine whether changes should be made. Ultimately, there is a need to regulate AI-based medical devices, and the SFDA has been bold in taking on this challenge. It will be critical to examine how its guidelines evolve and the extent to which other regulators align with this approach or forge a different path.

## Footnotes

1 The ‘AI to Law’ four stage process has been proposed elsewhere (Solaiman 2023b).

2 Also called AI/ML-enabled medical devices.

3 Personal Data Privacy Protection Law of 2016 (PDPPL).

4 For a more detailed analysis, see, Solaiman et al. (2024).

5 For a more normative appraisal of medical device regulations in this area, see, Gerke et al. (2020).

6 This paper refers to the official translated versions of the regulations available from the Saudi Food and Drug Authority.

7 The guidance uses the terms ‘developer’ and ‘manufacturer’ interchangeably in the MDS-G010.

8 Regulation (EU) 2017/746 of the European Parliament and of the Council of 5 April 2017 on In Vitro Diagnostic Medical Devices and Repealing Directive 98/79/EC and Commission Decision 2010/227/EU [2017] OJ L117.

9 Personal Data Privacy Protection Law of 2016 (PDPPL).

10 https://www.ansi.org/american-national-standards/ans-introduction/overview#introduction.
